# The Impact of β-Radiation Crosslinking on Flammability Properties of PA6 Modified by Commercially Available Flame-Retardant Additives

**DOI:** 10.3390/polym14153168

**Published:** 2022-08-03

**Authors:** Florian Tomiak, Dietmar Drummer

**Affiliations:** 1 Institute of Polymer Technology, Friedrich-Alexander-Universität Erlangen-Nürnberg, Am Weichselgarten 10, 91058 Erlangen, Germany; dietmar.drummer@fau.de; 2 Bavarian Polymer Institute, Friedrich-Alexander-Universität Erlangen-Nürnberg, Dr. Mack Strasse 77, 90762 Fuerth, Germany

**Keywords:** nylon 6, PA6, radiation crosslinking, fire testing

## Abstract

A comparative study was conducted investigating the influence of β-radiation crosslinking (β-RC) on the fire behavior of flame retardant-modified polyamide 6 (PA6). In order to provide a comprehensive overview, a variety of commercially available flame-retardant additives were investigated, exhibiting different flame retarding actions such as delusion, char formation, intumescence and flame poisoning. This study focused on the identification of differences in the influence of β-RC on fire behavior. Coupled thermal gravimetrical analysis (TGA) and Fourier transformation infrared spectroscopy (FTIR) were used to conduct changes within the decomposition processes. Dynamic thermal analysis (DTA) was used to identify structural stability limits and fire testing was conducted using the limiting oxygen index (LOI), vertical UL-94 and cone calorimeter testing. Crosslinking was found to substantially change the fire behavior observed, whereas the observed phenomena were exclusively physical for the given formulations studied: warpage, char residue destruction and anti-dripping. Despite these phenomena being observed for all β-RC formulations, the impact on fire resistivity properties were found to be very different. However, the overall fire protection properties measured in UL-94 fire tests were found to deteriorate for β-RC formulations. Only β-RC formulations based on PA6/EG were found to achieve a UL-94 V0 classification.

## 1. Introduction

β (e-beam) or γ radiation crosslinking (RC) provides substantial improvements in mechanical, thermal and chemical properties and enables standard or technical polymers to be used in technical applications that alternatively require high-performance polymers [1,2]. RC is known to enhance the polymer/polymer and polymer/particle bonding strength, resulting in improvements in welding bond strength, tensile strength, heat resistance and mechanical stability at higher temperatures (no melt dripping), as well as tribological properties, hydrolysis, ageing and stress cracking resistance [3,4,5,6,7,8]. In principle, all polymers can be radiation crosslinked, though some (polypropylene PP and polyamide PA) require the addition of crosslinking additives [9]. The radiation process is based on high-energy β or γ radiation impact, which leads to unsaturated bonds and the subsequent formation of an intermolecular RC network. Besides intended changes regarding mechanical, thermal and chemical properties, the network formation also has the potential to alter crucial fire behavior characteristics. There are only a few published studies to date that examine the influence of radiation crosslinking on the fire behavior of net, reinforced and flame retardant-modified polymers.

The majority of available studies examine the effect of radiation crosslinking on the fire behavior of cable isolation and jacketing materials such as PE, EVA or their corresponding blends, PE/EVA [10,11,12] or PE/EPDM [13,14]. This is little surprising since RC is commercially used in the electric and electronic industries, where products require multiple properties to be fulfilled for them to be put into use, such as mechanical, thermal and chemical properties, as well as highly flame-retardant classifications. Studies are available on the impact of the radiation crosslinking and flame retardant effects of filled (PE/EVA/Mg(OH)_2_ [11], reinforced PA6/PA6.6 systems [15], flame-retardant systems modified by red phosphorus (PA6 [16], PA6.6 [17] and PBT [18], and PET [19]) and PA6 containing ammonium polyphosphate (APP), melamine (MA) and phospham (PH) as flame-retardant additives [20]. Particularly promising flame retarding properties have been reported for silica additives, which are commercially used in crosslinked cable sheathing materials. Multiple studies are available that review the flame retardancy properties of kaolin, calcium carbonate, montmorillonite, wollastonite, etc., singularly or as synergists in EVA and PDMS matrices [21,22,23]. Most studies exhibit fire testing results from limiting oxygen index (LOI) and UL-94 as fire testing methods, whereas only some present the fire behavior in cone calorimeter testing setups (PP/PA6) [14,24].

It is generally understood that crosslinking reduces melt dripping phenomena, since the three-dimensional network retains its structural stability until decomposition occures [19]. This might be advantageous for applications where melt dripping must not occur, but can affect results in dripping-sensitive fire tests such as LOI and UL-94. For some polymers, such as PE, RC has been reported to increase the thermal stability and ignition temperature, which potentially improves temperature resistance and fire resistance properties [25]. One study has reported that crosslinking increases the charring tendency for some polymers such as PMMA due to crosslinking activities which occur during decomposition [26]. However, this is no general effect and does not necessarily occur for other polymers such as PA6.

Only a few studies are available assessing the effect of crosslinking on fire behavior provided by flame-retardant polymeric systems. Some phosphorus-containing (red phosphor (RP), PH, and APP) systems have been reported to increase char formation when crosslinked, indicating a change in chemical interactions. This was found to improve the UL-94 rating by at least one level. However, oxygen indices have not been found to necessarily follow this trend. Whereas formulations in PA6 and PA6.6 have been reported to show a clear improvement in OI values, by 4–5% (PA6 [16,20], PA6.6 [17]); measurements for PBT and PBT indicated no changes (PBT [18]; PET [19]). Other flame-retardant systems, such as melamine (MA), have been shown to deteriorate the OI. Thus, it can be concluded that the effects on flame retardancy properties are individual and cannot be generalized.

Another important property that has been reported to occur for RC samples is warpage. Warpage occurs due to strong asymmetric heat impact and subsequent temperature gradients throughout the sample diameter. Since RC materials exhibit good physical stability at high temperatures, internal stresses originating from temperature-dependent elongation behavior can only be eliminated by deformation. Warpage has been reported to be particularly disadvantageous for char forming flame-retardant additives. Freshly formed char residue is frequently disrupted, preventing a sufficient barrier from being built up [24].

Besides the reported changes in the functionality of fire safety properties, it is also worth mentioning that crosslinking processes might also be affected by the presence of flame-retardant additives. For PA6 containing ammonium polyphosphate (APP), melamine (MA) and phospham (PH), a study has shown substantial differences in the resulting gel fractions after radiation, depending on the flame-retardant additive present. All three flame-retardant additives appeared to either accelerate or decelerate the radiation process. Accordingly, to gain a fully crosslinked system, the normally sufficient radiation dosage of 100 KGy for PA6 had to be adjusted by a factor between 0.8 and 3 [20].

The literature provides a wide variety of findings regarding the effect of radiation crosslinking on flame-retarding properties, though lacks a general assessment of how different acting flame-retardant additives effect the burning behavior in modern fire testing setups. The available studies focus on individual effects with regard to fire behavior in specific testing setups, though do not provide a general overview on how radiation crosslinking affects important flame-retarding mechanisms, such as intumescence, gas phase dilution, melt flow and dripping, cooling effects and flame poisoning.

Within this study we investigated the fire behavior of a variety of commercially available flame-retardant additives at systematically varied filling degrees in non- and β-RC PA6. The flame-retardant additives used were selected in order to provide a wide variety of flame-retarding mechanisms. Triallyl Isocyanurate (TAIC) was used as a crosslinking additive. Crosslinking was carried out by β-radiation and a dosage of 3 × 33 kGy, which is standard for PA6/TAIC formulations. Thermal measurements were conducted using DSC and coupled TGA–FTIR analysis to investigate thermal and decomposition characteristics. LOI, UL-94 V and cone calorimeter tests were performed to assess flame inhibition mechanisms and torsion DMA. This study provides comprehensive results regarding the fire behavior of β-RC PA6 modified by different flame-retardant additives. Care is taken to explain and evaluate the effects that influence the fire behavior due to crosslinking.

## 2. Materials and Methods

### 2.1. Materials and Preparation

A standard PA6 grade (PA6 B27E; density: 1.13 g/cm^3^; MVR: 130 cm^3^/10 min at 275 °C/5 kg) from BASF SE (Ludwigshafen, Germany), an expandable graphite GHL PX 95 HT 270 (70% > 50 mesh; pH: 5–9) from LUH GmbH (Walluf, Germany), melamine cyanurat MC50 (98% < 25 µm; pH: 5–6) from BASF SE (Ludwigshafen, Germany), Exolit OP 1314 (phosphorus, 20.5–21.5%) and an Exolit OP 1230 (density: 1.35 g/cm^3^; d_50_: 20–40 µm; phosphorus: 23.3–24.0%; low water solubility) from Clariant AG (Muttenz, Switzerland) were used in this study. As the radiation crosslinking additive, we used the masterbatch material TAICROS PA6 60 (TAIC) from Evonik Industries AG (Essen, Germany). Key figures were obtained from data sheets. All flame-retardant additives were separately compounded into the PA6 matrix using systematically varied filling degrees, listed in Table 1.

Compounds were produced using a twin-screw extruder (co-rotating) DSE ZSE HP 27 from Leistritz GmbH (Nuremberg, Germany), operating two gravimetrical feeder units. The strand was drawn off through a water bath, chipped to granulate and dried afterwards for further processing. For sample preparation all compounds were injection molded into plates with geometries of 115 × 115 × 1 and 4 mm^3^ using an injection molding machine Arburg Allrounder 370 V from Arburg GmbH and CoKG (Loßburg, Germany). Aggregate temperatures were controlled between 230 °C (die) and 220 °C; injection speed was 60 mm/s; mold temperature was 80 °C. Materials prepared for further radiation crosslinking were additionally dry-blended with 3 wt.% TAIC, corresponding roughly to 2 wt.% crosslinking agent present within the recipes. β-radiation crosslinking was performed using a 3×33 KGy radiation dose under room temperature and ambient conditions. Samples did not exceed a peak temperature of 50°C throughout the radiation process. Sample geometries were prepared by sawing and milling. The geometries prepared were as follows: cone calorimeter samples—100 × 100 × 4 mm; LOI samples—125 × 10 × 1 mm; UL-94 V—125 × 13 × 1 mm. Samples were subjected to dry (70 °C at reduced pressure) and humid (60% rel. humidity, 70 °C) conditions, until the same weight was achieved.

### 2.2. Degree of Crosslinking

To determine the degree of crosslinking of PA6, crosslinked recipes containing 20 wt.% of a flame-retardant additive were chemically solved for 6 h (120 °C) in a 98% formic acid solution. The gel fraction was then filtered (16 µm) and washed with distilled water until reaching a neutral pH value. Since incineration does not work for flame-retardant additives to determine the residual fraction within the non-gel and gel fractions, a 20 wt.% filler content was assumed. As a result, the “true” gel content might therefore be slightly lower. All tests were repeated three times.

### 2.3. Thermal and Gas Analytics

Coupled thermogravimetric analysis (TGA) and Fourier transform infrared spectroscopy (FTIR) were conducted under nitrogen atmosphere to identify potential changes in decomposition modes that might occur due to crosslinking. TGA measurements were conducted at a heating rate of 20 K/min between 50 and 800 °C and 70 mL nitrogen flow. The onset temperature is defined as 99% residual mass. All tests were conducted three times, and averaged curves are presented. Sample weights were kept constant at 10 ± 1 mg. TGA and DSC analysis were conducted using a simultaneous scanning analysis device, STA F3 449 Jupiter, from Netzsch (Selb, Germany). FTIR gas analysis was performed simultaneously to TGA measuring using direct coupling through a 230 °C temperature-controlled transfer line. The FTIR unit was a Tensor 2 from Bruker Corp. (Billerica, MA, USA). FTIR gas-cell temperatures were controlled at 200 °C; 32 scans were averaged. The gas transfer refers to a 30 s measurement delay between the TGA and FTIR results, which corresponds to 10 °C (20 K/min).

Additionally, TGA measurements were used to calculate decomposition activation energies using the methods described by Vyazovkin [27] and Ozawa [28]. The heating rates used were 2.5, 5 and 10 K/min, under nitrogen atmosphere.

### 2.4. Fire Testing

Fire testing was conducted using a cone calorimeter (ISO 5660-1) as well as UL-94 (DIN EN 60695-11-10/20) and LOI (DIN EN ISO 4589-2) fire tests. All testing devices were from Netzsch Taurus GmbH (Weimar, Germany). The samples were dried at reduced pressure at 70 °C until the weights were consistent.

Cone calorimeters provide a laboratory-scale testing setup under enforced flaming conditions and can give us important insights of the fire behavior throughout ignition, flame spread and full burning stages. The most important key figures are the heat release rate (HRR), the peak heat release rate (pHRR), total heat emitted (THE), time of ignition (t_ign_), smoke production rate (SPR), total smoke production (TSP), average specific extinction area (ASEA), mass loss rate (MLR), and average mass loss rate (AMLR). Tests were conducted under multiple heat fluxes, 35, 50 and 65 kW/m^2^, to gain information about the heat response characteristics. All tests were repeated at least two times.

The limiting oxygen index (LOI) was used to evaluate the ignitability of polymeric materials and it provides a continuous key figure that is particularly useful for trend analysis. The oxygen index indicates the oxygen content necessary in order to provide appropriate atmospheric conditions for a visible flame to spread down the sample. Small changes within the material recipe can thus be evaluated in terms of ignitability.

Vertical UL-94 fire testing is used to evaluate the self-extinguishing properties of polymeric materials. The test provides an observation-based evaluation system that allows one to categorize self-extinguishing and dripping behaviors using a normalized classification scheme. Classifications provided by vertical UL-94 tests are V0, V1, V2 and HB, which were then assigned to a set of observations. V0-classified materials showed almost immediate self-extinction after the test flame was removed and no burn dripping. V2 classifications allow longer burning times as well as burn dripping. V1 classifications represent an intermediate state between V0 and V2, allowing longer burning times but no burn dripping. All tests conducted within this study were performed in accordance with the standards.

### 2.5. Char Residue Analysis

The Char residue analysis focusses on the discussion of differences found for non-RC and RC samples tested under cone calorimeter conditions. Images are provided showing the cross section of cone calorimeter samples after 120 s. Samples were therefore extracted from the ongoing testing process, extinguished and cooled until solidification. In order to cause as little damage as possible, samples were than broken in the mid-section. The residual char after completion was also optically analyzed. Visible changes between non-crosslinked and crosslinked samples were discussed and referenced to observed fire behavior during cone calorimeter testing.

### 2.6. Dynamic Mechanical Analysis (DMA)

Torsion dynamic mechanical analysis was performed using an analysis device, Ares, from TA Instruments (New Castle, DE, USA) at 1 Hz and 0.05% strain. The method was used to gain information about the physical material stability at elevated temperatures. Tests were conducted between 40 °C and 300 °C at a heating rate of 3 K/min under normal atmospheric conditions. When non-crosslinked samples exceeded melting temperatures, the measurements had to be stopped due to the appearance of strong melt flow. Sample geometry used was 10 × 40 × 4 mm^2^.

## 3. Results

### 3.1. Degree of Radiation Crosslinking—Cristallization Behavior and Gel Content

**Figure 1:**
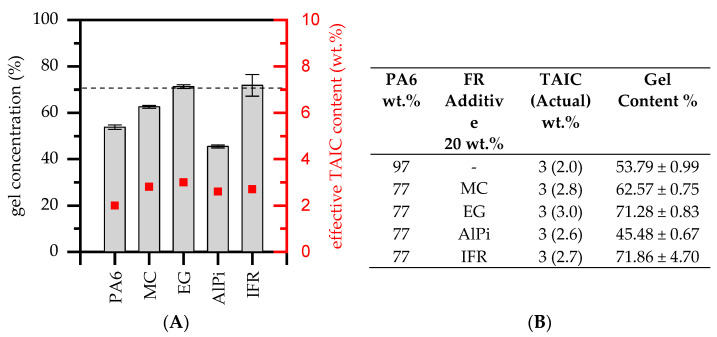
(**A**) Gel content measured as reference to the degree of crosslinking. The effective TAIC content was calculated from the PA6 fraction present within each formulation. (**B**) Summary of gel and actual TAIC contents.

To evaluate the degree of crosslinking, an evaluation of gel content was conducted. Depending on the formulation, the gel contents measured were between 54% and 72%. Literature values propose the gel content of PA6 modified by TAIC to be around a maximum of 70% for a β-radiation intensity of 3×33 KGy [29]. Since the TAIC type used contains 60% TAIC (40% PA6) and was incorporated by dry blending during the injection molding process, the effective dosage of TAIC varied between 2.0 wt.% (PA6) and 2.8 wt.% (PA6/IFR). The exact amounts of TAIC within each formulation are provided in Figure 1.

Despite large discrepancies of the resulting gel contents, some flame-retardant additives seemed to substantially impact the crosslinking process. The lowest TAIC fraction was incorporated into net PA6 (2 wt.%), exhibiting a gel content after β-RC of about 54%. This comparably low gel content can be attributed to the TAIC content of about 2.0%, which was not high enough to achieve the values reported in the literature. Formulations containing EG and IFR, containing relative TAIC loads of 3.0% and 2.7%, were measured to have a higher gel content of about 72%. This can be attributed to an overall higher TAIC load. However, formulations containing identical filling degrees of MC and AlPi, as well as comparable TAIC weight fractions, exhibited substantially lower gel fractions of 63% and 45%, respectively. Similar trends have been reported in the literature, whereas some flame-retardant additives seemed to interact with the crosslinking process, resulting in lower gel formation [20]. However, a concrete explanation cannot be given. Further studies are needed in order to identify the interaction modes of gel formation provided by the given flame-retardant additives.

### 3.2. Thermal Decomposition Behavior

#### 3.2.1. Thermal Decomposition Behavior—PA6

**Figure 2:**
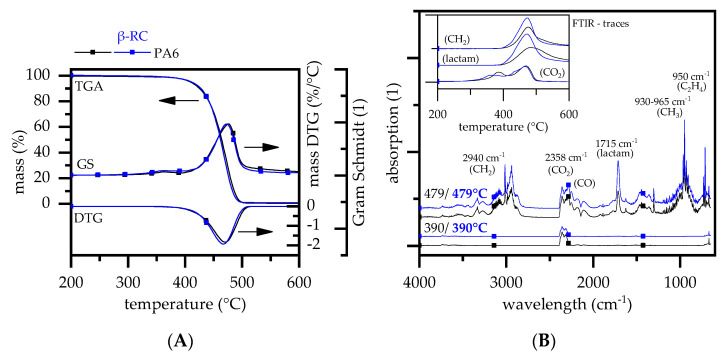
TGA, DTG, Gram Schmidt and FTIR (Gas) curves determined at a heating rate of 20 K/min. (**A**) TGA, DTG Gram Schmidt for net PA6; (**B**) corresponding FTIR peak spectra.

The degradation process of non-β-RC PA6 has been widely studied in the literature [30,31,32,33]. When measured under steady heating conditions and nitrogen atmosphere, PA6 is known to degrade in a single decomposition step at characteristic temperatures between 400 °C and 500 °C, with only little char formation (1.7%) [34]. This fits well with the findings within this study, revealing a decomposition onset temperature (T_99%_) at 353 °C/(T_90%_) of 425 °C, DTG peak at 479 °C and completed combustion at 504 °C (Figure 2A).

PA6 is reported to mainly decompose to its monomer caprolactam and subsequent volatile decomposition products [35]. An additional, non-gravimetrical decomposition peak at 390 °C only releasing CO_2_ could also be found in FTIR gas analytics. The early CO_2_ release is reported to originate from hydrolytic scission of the C(O)-NH bond and the subsequent decomposition of carbon acid to CO_2_, CO and water [36,37]. The main decomposition step, marking a sharp gravimetrical decrease around 479 °C, is reported to evolve the following volatile decomposition products measured in FTIR analysis [38,39,40]: caprolactam (1715 cm^−1^; fingerprint pattern: 1305, 1352, 1361 and 2865 cm^−1^), ammonia NH_3_ (930 and 965 cm^−1^), other NH groups (3334 cm^−1^), CH2 groups (2940, 2865, and 1440 cm^−1^), ethene (950 cm^−1^), methane (3015 cm^−1^; fingerprint pattern: 1200–1400 cm^−1^), CO_2_ (2360 and 671 cm^−1^), CO (2114 and 2174 cm^−1^) and water (3853; fingerprint pattern: 3400–4000 cm^−1^). All characteristic FTIR peaks were also found within this study.

β-RC PA6 also decomposed within a single step [41,42]. The onset temperatures were found to be slightly higher than non-β-RC PA6. The activation energies calculated for non-β-RC PA6 and β-RC PA6 using the calculation methods introduced by Ozawa differed slightly, with 218 kJ/mol (Ozawa) and 210 kJ/mol (Ozawa) for non-β-RC PA6 and β-RC PA6, respectively. This fits well with the values reported in the literature (170 kJ/mol–230 kJ/mol) [33,36,43]. The lower activation energy of β-RC PA6 can be attributed to the crosslinking process. Crosslinking includes the cracking and rearrangement of molecular chains, chemically linking to neighbor polymers and thus forming a three-dimensional network. A certain fraction of cracked molecules cannot be rearranged and remain as a residual monomer. This reduces the amount of intermolecular links relative to the non-crosslinked system, which, as a consequence, reduces the activation energy [44].

The DTG peak occurred slightly earlier for β-RC PA6, indicating an accelerated decomposition processes at higher temperatures. FTIR gas phase analysis showed no changes regarding the chemical gas phase composition for β-RC PA6, but indicated a temperature shift and intensity change of some evaporated gas phase fractions over time (Figure 2B). CO_2_ was found to evaporate slightly earlier for β-RC PA6 within the first non-gravimetrical decomposition step and indicated an evaporation peak at lower temperatures. Caprolactam (1715 cm^−1^) and subsequent decomposition products containing CH_2_ (2940, 2865, and 1440 cm^−1^) groups were found to evaporate more intensively during the second decomposition step, as shown by the a sharper evaporation and a slightly earlier peak. Further studies are needed to provide clear reasoning for this effect.

The residue was found to be 0.7%, which is slightly lower than that measured for non-β-RC PA6. Some studies have reported a slight increase in residual fractions after radiation crosslinking, which could not be confirmed for the β-RC PA6 tested within this study [20,45].

#### 3.2.2. Thermal Decomposition Behavior—PA6 Containing MC

**Figure 3:**
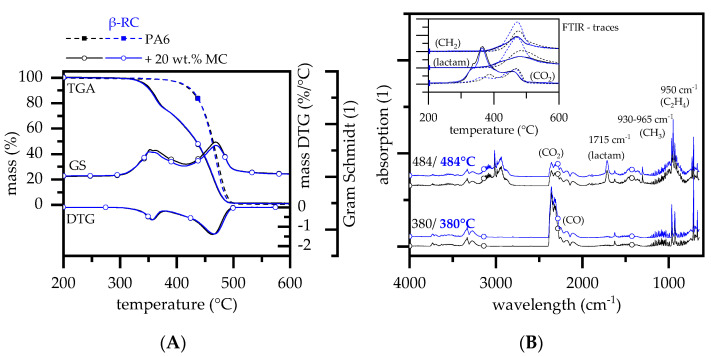
TGA, DTG, Gram Schmidt and FTIR (Gas) curves determined at a heating rate of 20 K/min. (**A**) TGA, DTG Gram Schmidt for PA6 containing 20 wt.% MC; (**B**) corresponding FTIR peak spectra.

Melamine cyanurate (MC) has been widely studied as flame-retardant additive in PA6. The modes of action can be summarized as endothermal cooling and the gas phase dilution effect. Studies have shown that PA6 containing MC decomposes in two major gravimetrical steps evident in TGA analysis, whereas the onset temperature is substantially lower than for net PA6. The first decomposition step is solely dedicated to MC decomposition, with no known interaction between PA6 and MC, whereas the second decomposition step is reported to be mainly PA6 [46,47,48]. This fits well with the findings in our study. TGA measurements for non-β-RC PA6 containing 20 wt.% MC revealed two gravimetrical decomposition steps with a degradation onset temperature of 320°C, DTG peaks at 357 °C and 466 °C (Figure 3) and no substantial residual formation (1 ± 0.4%). Activision energies calculated for the first and second steps (Ozawa) were 194/170 kJ/mol (non-β-RC PA6/MC) and 221/177 kJ/mol (β-RC PA6/MC). This implies changes in reactivity processes, accelerating decomposition reactions for non-β-RC PA6/MC and decelerating ones for β-RC PA6/MC material combinations.

The first gravimetrical decomposition step revealed the evaporation of ammonia NH_3_ (930 + 965 cm^−1^) and other NH groups (3334 cm^−1^), CO (2183 cm^−1^), CO_2_ (2358 cm^−1^), and ethane, C_2_H_4_ (950 cm^−1^), which can be due to MC decomposition and an early dehydration mode of primary amide chain ends [38,39,40,49]. The second step at 484 °C corresponds to the main gravimetrical step seen in TGA measurements at 477 °C, considering a slight time gap between pyrolysis and the FTIR gas chamber. The gas phase composition was found to be chemically identical to net PA6, mostly evaporating caprolactam (1715 cm^−1^), ammonia NH_3_ (930 and 965 cm^−1^) and other NH groups (3334 cm^−1^), CH2 groups (2940, 2865, and 1440 cm^−1^), ethene (950 cm^−1^), methane (3015 cm^−1^), CO_2_ (2360 and 671 cm^−1^), CO (2114 and 2174 cm^−1^) and water (3853 and 3400–4000 cm^−1^). Slightly lower absorption intensities found at 484°C can be attributed to a lower residual fraction, since a substantial weight fraction has already been consumed. TGA-FTIR measurements of β-RC PA6 containing 20 wt.% MC revealed an identical decomposition process, both gravimetrically and chemically.

#### 3.2.3. Thermal Decomposition Behavior—PA6 Containing EG

**Figure 4:**
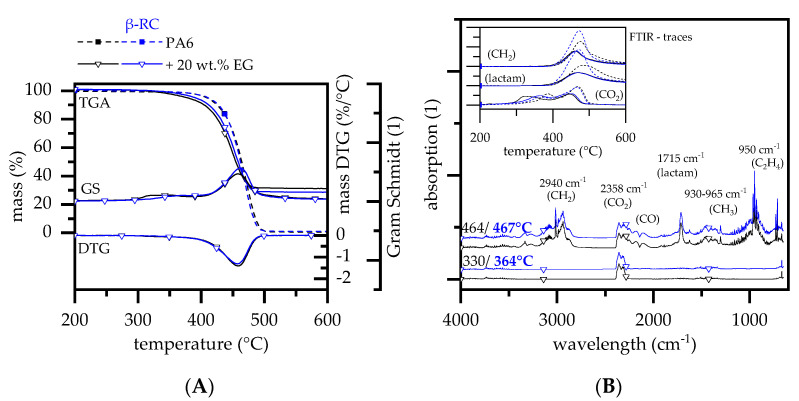
TGA, DTG, Gram Schmidt and FTIR (Gas) curves determined at a heating rate of 20 K/min. (**A**) TGA, DTG Gram Schmidt for PA6 containing 20 wt.% EG; (**B**) corresponding FTIR peak spectra.

The decomposition behavior of non-β-RC PA6 containing expandable graphite (EG) has been widely studied in the literature [50,51,52]. When a critical temperature is reached, incorporated EG acts solely through expansion and residue formation, providing a thermally stable and voluminous heat barrier on the sample surface. Multiple studies have been published using EG in a variety of different polymeric matrices as a flame-retardant additive (z. B. PP [53,54,55,56,57,58,59], PE [60,61,62,63], ABS [64], EVA [65,66], PET [67,68], PS [69], PVC [70]). No chemical interactions between EG and any polymeric matrix have been reported to be relevant for the flame-retardant effect. Accordingly, it is generally accepted that flame-retardant properties are mainly dependent on the physical expansion behavior, including the residual built-up speed and final residue volume [71,72]. For non-β-RC PA6 containing 20 wt.% EG, decomposition predominantly occurred in a singular gravimetrical step, marking a DTG peak at 460 °C for non-β-RC (Figure 4A). Activision energies calculated (Ozawa) were 169 kJ/mol (non-β-RC PA6) and 180 kJ/mol (β-RC PA6). The decline in activation energies can be attributed to an overlaying effect of PA6 decomposition and EG expansion, which gradually evolves a gaseous blowing agent.

A slight difference to non-β-RC net PA6, marking a DTG peak at 468°, was attributed to the expansion effect of EG, which increased the surface area and provided a larger evaporation zone. Due to the early reaction mode of EG, a second decomposition step was found at a FTIR peak temperature of 330 °C (Figure 4B). It has been reported that the early decomposition mode can be attributed to the decomposition of the intercalated sulfuric blowing agent [50]. As postulated in [71,73], the sulfur-containing agent (H_2_SO_4_) reacts by a redox reaction with graphitic carbon to form CO_2_, SO_2_ and water. The formation of CO_2_ and SO_2_ provides a density change between graphene layers and thus initiates the graphene layers to expand, eventually forming a typical worm-like structure. FTIR analysis revealed mostly CO_2_ evaporation around 330 °C as well as traces of SO_2_ and water. SO_2_ was found to predominantly evaporate at higher temperatures exceeding well over 360 °C, which fits well to findings reported in other studies [52].

When comparing non- and β-RC PA6 containing 20 wt.% EG, decomposition curves were mostly identical, with a single main gravimetrical decomposition step found at 460 °C. The decomposition onset temperature (T_99%_) was slightly higher at 350 °C (for non-β-RC PA6 containing 20 wt.% EG: T_99%_ 333 °C), with only little effects on the subsequent TGA decomposition characteristic. A slightly higher decomposition onset temperature was attributed to a shift in CO_2_ evaporation. This is caused by a more limited intra-material gas transfer, given by the three-dimensional crosslinked network in β-RC samples. Thus, no melting occurs and the structural stability is maintained until decomposition temperatures are reached.

#### 3.2.4. Thermal Decomposition Behavior—PA6 Containing AlPi

**Figure 5:**
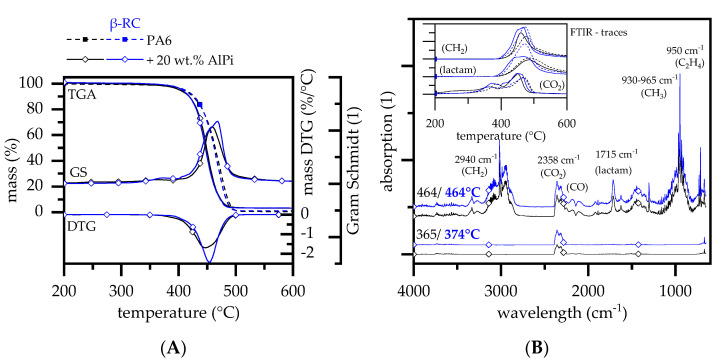
TGA, DTG, Gram Schmidt and FTIR (Gas) curves determined at a heating rate of 20 K/min. (**A**) TGA, DTG Gram Schmidt for PA6 containing 20 wt.% AlPi; (**B**) corresponding FTIR peak spectra.

AlPi has widely been studied as flame-retardant additive in a variety of polyamide types such as PA6 [74], PA66 [75] or PA6T [76]. It is generally accepted that the flame-retardant effect of AlPi can follow two reaction pathways, which may vary in dominances depending on the polymer matrix present. Incorporated in PA6, AlPi has been reported to act both in the gas phase by flame inhibition and in the mesophase by phosphoric char formation [74]. AlPi is characterized by a high thermal stability. TGA measurements for non-β-RC PA6 containing 20 wt.% AlPi thus showed a single decomposition step characteristic, marking, compared to net PA6, a slightly lower decomposition onset temperature of 357 °C and DTG peak temperature of 447 °C (Figure 5A). Activision energies calculated (Ozawa) were 222 kJ/mol (non-β-RC PA6/AlPi) and 234 kJ/mol (β-RC PA6/AlPi), which are thus slightly higher than calculated for net PA6. This effect can be attributed to the char-forming tendency of PA6/AlPi.

The residual fraction was also slightly higher at 3.8%, which can be attributed to aluminum phosphate and carbonaceous char formation [74]. A second, non-gravimetrical decomposition step was also identified within FTIR gas measurements. Similar to net PA6, FTIR measurements indicated an early CO_2_ evaporation around 365 °C, originated from early hydrolytic scission of the C(O)-NH bond as described earlier. FTIR bands for phosphinic acid (855 cm^−1^) and phosphinate (1146 cm^−1^) as described in [74] could not be clearly identified, most likely due to interfering bands.

β-RC PA6 containing 20 wt.% AlPi decomposed similar to non-β-RC PA6, following an identical gravimetrical mass loss characteristic in TGA measurements and indicated no changes within the chemical gas phase composition. However, some gas phase components showed an alteration in the quantitative presence, as indicated by a change in absorption-temperature profiles (Figure 5B). The observed evaporation rate of CO_2_ was initially lower for β-RC samples and quickly increased at elevated temperatures. A similar effect was identified for previously discussed formulations, which was explained by the transportation limits of pyrolysis gases caused by the physical stability of the crosslinked network. Caprolactam and CH_2_ containing molecular fractions, on the other hand, seemed to evaporate at lower temperatures and at a higher evaporation rate for β-RC compared to non-β-RC samples. A similar behavior was identified for net PA6. Since the residual fraction in TGA measurements did not change, it is suggested that an earlier evaporation mode of caprolactam and subsequent CH_2_-containing molecules is provided by a lack of intermediate crosslinking, apparent for the non-β-RC samples. Crosslinking provides an intermediate char residue, which was only somewhat temperature-stable and thus decomposed at higher temperatures. However, an intermediate char formation shifts the evaporation process of large caprolactam fragments to higher temperatures. This effect might occur in parallel to phosphorus charring.

#### 3.2.5. Thermal Decomposition Behavior—PA6 Containing an IFR

The IFR used within this study is a commercially available multi-material flame-retardant system, which contains mainly hypophosphite and melamine polyphosphate [77]. Details regarding further synergistic additives used within the recipe are not publicly available, which limits the following discussion. Since the IFR provided an additional class of flame-retardant mechanisms and exhibited good flame-retarding properties for PA6, it is worthwhile to include within the discussion.

**Figure 6:**
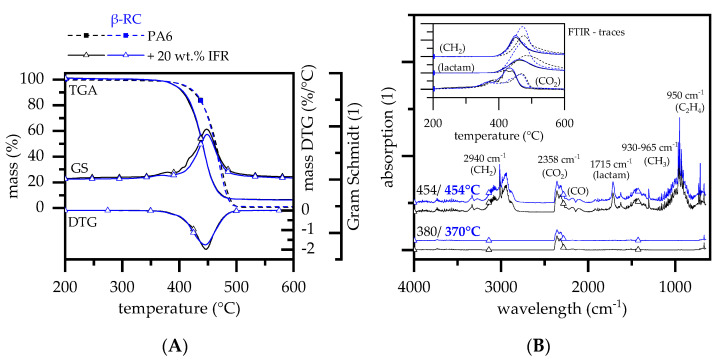
TGA, DTG, Gram Schmidt and FTIR (Gas) curves determined at a heating rate of 20 K/min. (**A**) TGA, DTG Gram Schmidt for PA6 containing 20 wt.% IFR; (**B**) corresponding FTIR peak spectra.

TGA measurements of non-β-RC PA6 containing 20 wt.% IFR revealed a single-step decomposition process, marking an onset temperature at 369 °C and a DTG peak at 445 °C (Figure 6A). FTIR analysis revealed a non-gravimetrical decomposition step at a peak temperature of 380 °C, mostly evolving CO_2_. A similar effect was identified for all formulations tested. Gas phase products found within the main decomposition step did not indicate changes compared to net PA6. However, the amount of particular gas phase products varied over time (Figure 6B). The activation energies calculated (Ozawa) were 230 kJ/mol (non-β-RC PA6) and 227 kJ/mol (β-RC PA6).

Initial CO_2_ evaporation of non-β-RC PA6 containing 20 wt.% IFR was detected at similar temperatures, as observed for non-β-RC net PA6. Subsequently, the quantitative evolution accelerated quickly, marking a CO_2_ evaporation peak at around 430 °C. Compared to an according peak at 470 °C identified for non-β-RC net PA6, CO_2_ evaporation was intensified due to the presence of the multi-material flame-retardant mixture. This effect was attributed to the decomposition process of a blowing agent. Caprolactam and its decomposition products containing CH_2_ showed similar evaporation onset temperatures and initial evaporation quantities compared to net PA6. However, the overall evaporation rate was found to be lower and evaporation peaks were reached at substantially lower temperatures. Since non-β-RC PA6 containing 20 wt.% IFR showed stronger residual formation within TGA (~5.5 wt.%), the lower evaporation rate of caprolactam was attributed to increased charring, which in turn effectively bound and solidified larger caprolactam fractions.

β-RC PA6 containing 20 wt.% IFR was found to behave mostly identical. TGA characteristics and FITR gas phase fragments were identical. Only some variations occurred within the first non-gravimetrical decomposition step; CO_2_ evaporation was found to start at slightly lower temperatures. A similar effect could be identified for non-β-RC and β-RC net PA6, though a specific explanation cannot be given.

#### 3.2.6. Thermal Decomposition Behavior—Summary

Coupled TGA and FTIR gas analysis were conducted in order to investigate the decomposition behavior of non-β-RC and β-RC PA6. The investigation was carried out using four types of commercially available flame-retardant additives, which have been incorporated into a PA6 matrix. Each flame-retardant additive provided an individual mechanism to improve the burning behavior.

This study identified no substantial changes within reaction pathways between non-β-RC and β-RC materials. Gravimetrical decomposition characteristics were mostly identical, showing only in some cases variations in the decomposition onset temperature. Shifts did not occur systematically for non-β-RC and β-RC materials but rather seemed to be dependent on the individual formulation. FTIR gas analysis revealed no changes within the chemical gas phase composition that would indicate alternative reaction pathways. However, net PA6 and formulations containing PA6 and AlPi showed a substantial change within the quantitative evolution profile of caprolactam. Caprolactam started to evaporate from temperatures around 400 °C and intensified its gas phase presence over subsequently rising decomposition temperatures. β-RC compared to non-β-RC materials seemed to evolve larger caprolactam fractions at a higher evaporation rate, marking an earlier and higher leveled absorbance peak as well as a subsequent steeper decline. Clear reasoning for this effect cannot be provided and so it should thus be researched in further studies.

**Figure 7:**
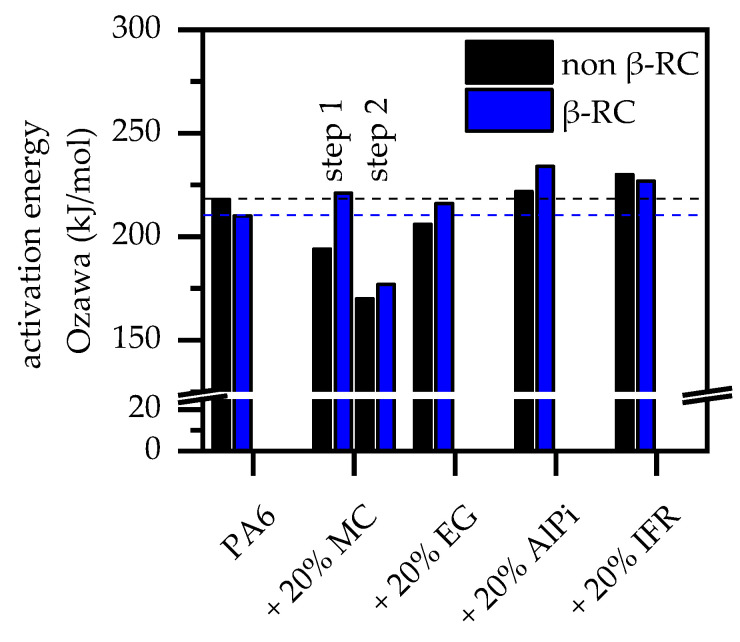
Activation energy evaluated for net PA6 and PA6 containing 20 wt.% MC, EG, AlPi, and IFR were calculated using the model introduced by Ozawa. TGA measurements were conducted at 2.5, 5 and 10 K/min under nitrogen atmosphere.

Although no obvious changes in the gravimetric decomposition characteristics and chemical gas phase products could be identified, the evaluation of the activation energy for non-β-RC and β-RC formulations showed significant differences (Figure 7). The activation energy decreased for net PA6 and PA6/IFR and increased for PA6/MC, PA6/EG, PA6/AlPi formulations when β-RC. Since this effect does not follow a particular trend, changes are attributed to formulation-specific changes within the mesophase decomposition process. Further studies are needed to clarify this effect. All TGA results are summarized in Table 2.

### 3.3. Dynamic Mechanic Analysis

**Figure 8:**
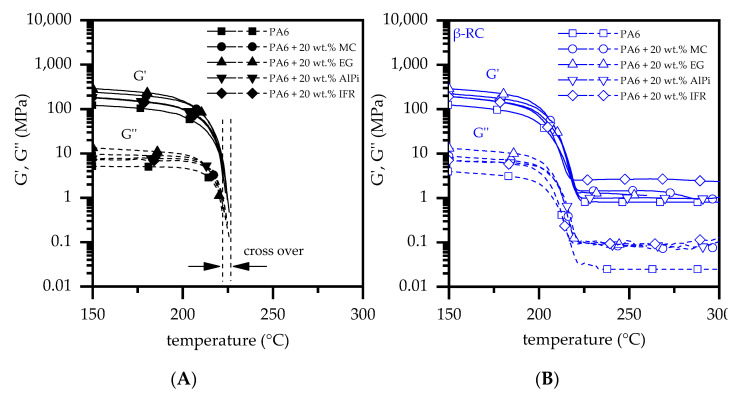
Torsion DMA results representing storage (G′) and loss (G″) moduli for (**A**) non-β-RC samples and (**B**) β-RC samples; 1 Hz; 0.05% strain.

Torsion DMA measurements were conducted in order to investigate the physical stability of all material formulations at elevated temperatures. Non-β-RC materials showed very similar mechanical characteristics, which exhibited a high level of physical stability until 200 °C (Figure 8A). A subsequent increase in temperatures revealed a sudden decrease in mechanical strength as well as a total loss in structural stability when the temperatures approached the melting point of PA6. The transition between solid and liquid is marked by a crossover between storage (G′) and loss modulus (G″), which is evident between 215 and 230 °C. As a consequence of the structural stability loss, strong melt flow occurred and characteristic geometry properties are eliminated. Starting from comparable levels of stiffness, β-RC materials showed a similar decrease in structural stability when melting temperatures were approached (Figure 8B). However, due to the intermolecular network structure, a certain stability level is maintained beyond the melting point. Since the storage module (G′) remains in a stable, second Plateau above the loss module (G″), no melt flow occurred and geometrical properties were maintained in most cases up to temperatures of 300 °C (measuring range). For formulations containing EG and MC, the structural stability deteriorated earlier, since the flame-retardant additives present started to decompose at 260 °C and 280 °C, respectively. However, no melt flow occurred, revealing structural stability somewhat beyond the individual reaction temperature. Similar characteristics have been reported in the literature [3,24].

**Figure 9:**
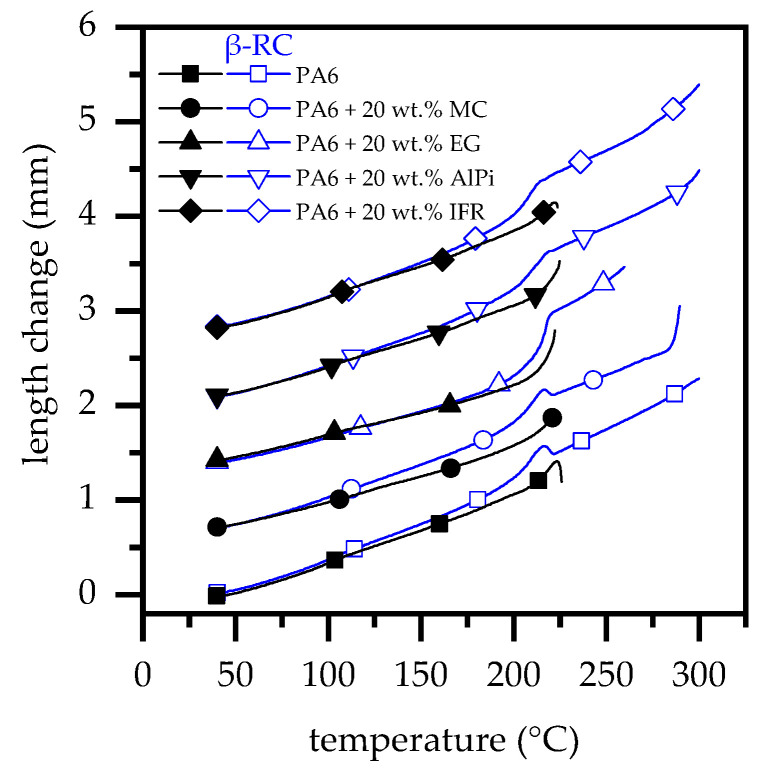
Longitudinal length change tracked during torsion DMA measurements for β-RC samples; 1 Hz; 0.05% strain.

The potential warpage effect becomes clearly evident through the observation of the temperature-dependent longitudinal length changes tracked during torsion DMA measurements (Figure 9). Up to the melting point, the non-β-RC samples stretch between 1.1 and 1.4 mm, which corresponds to a change in length of 5% to 7%.

By maintaining the structural stability of β-RC samples beyond the melting point, a further change in longitudinal growth can be observed. This amounts for samples containing MC, AlPi or IFR as flame-retardant additive to a total of 2.3 to 2.6 mm, which corresponds to a total longitudinal change of 9% to 11%. EG-containing formulations amount to 2.1 mm, which corresponds to 9%.

### 3.4. Fire Behavior—Cone Calorimeter Testing

#### 3.4.1. Cone Calorimeter Testing—PA6

Cone calorimeter tests were conducted at external heat fluxes of 35, 50 and 65 kW/m^2^ for non-β-radiation and β-radiation β-RC samples. The following chapter focuses on differences in the fire behavior found for flame-retardant non- and β-RC PA6 formulations. All flame-retardant additives tested are commercially available and have been extensively studied in the literature (MC [46,47,78], EG [50,51,52,79], AlPi [74,75], and IFR [80]). Thus, where appropriate, literature for further information is presented.

**Figure 10:**
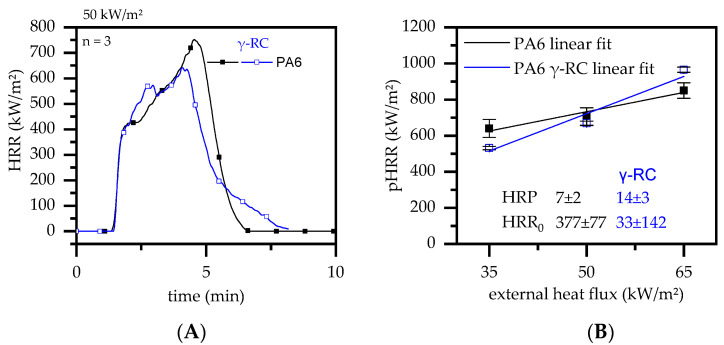
(**A**) HRR curves conducted by cone calorimeter testing for net and β-radiation β-RC PA6 at 50 kW/m^2^. (**B**) pHRR conducted by cone calorimeter tests at an external heat flux of 35, 50 and 65 kW/m^2^. A linear fit was used to evaluate the HRP and corresponding y-cross point HRR_0_.

Once ignited, PA6 is known for its rapid heat development showing a characteristically high pHRR and, since most of the fuel has been burned at this burning stage, a subsequent sharp decrease in heat development (Figure 10A).

A similar burning characteristic was evident for β-radiation β-RC PA6. The time to ignition (tign) was found to be identical for β-radiation β-RC PA6, whereas the pHRR and THE were slightly lower. Since the decomposition process was found to be identical, changes within the burning process can be attributed to different physical behavior. Non-β-RC PA6 tended to melt quickly, with the pyrolysis process taking place over the entire sample cross section. β-RC PA6 revealed an increased physical stability throughout the burning process. As a consequence, the pyrolysis process is more limited to areas close to the surface, resulting in a lower mass loss rate and thus a lower mass transport towards the combustion zone. This effect was found to be particularly evident for cone calorimeter tests conducted at 35 and 50 kW/m^2^, though seemed to be reversed for an external heat flux of 65 kW/m^2^ (Figure 10B). During the heating process, high amounts of energy were applied to the surface areas, which were then dissipated via thermal conductivity throughout the sample. The heat dissipation generates temperature gradients, with high temperatures close to the surface and lower temperatures at the bottom of the sample. Since temperature changes result in a linear expansion of materials, the hotter surface areas expand more quickly than the colder bottom areas. As described earlier, β-RC PA6 is physically more stable throughout the burning process than non-β-RC PA6, such that internal stress triggered through differing linear expansion rates results in physical warpage. Warpage was evident for all the external heat fluxes tested, though tended to accelerate when higher heat fluxes were applied. Since warpage occurs in a way that reduces the proximity between the sample surface and cone heater, the heat impact increases, followed by subsequent accelerated decomposition processes and higher pyrolysis gas streams, fueling the flame. As a result, β-RC PA6 was found to be more sensitive towards external heat flux changes (HRP: 14 ± 3) due to physical warpage compared to non-β-RC PA6 (HRP: 7 ± 2).

#### 3.4.2. Cone Calorimeter Testing—MC

**Figure 11:**
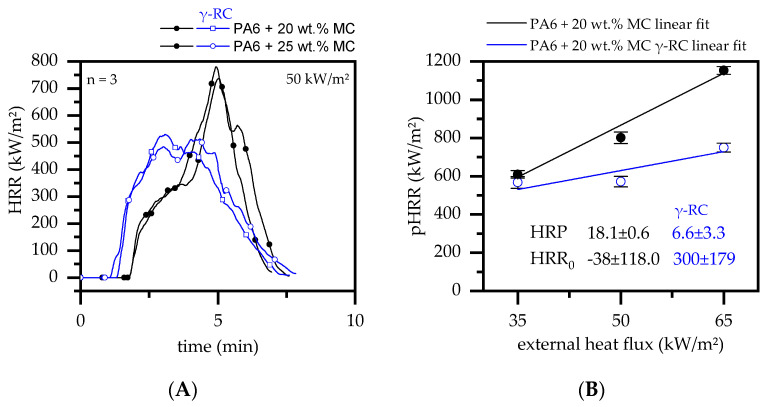
(**A**) HRR curves conducted by cone calorimeter testing for net and β-radiation β-RC PA6/MC formulations at 50 kW/m^2^. (**B**) pHRR conducted by cone calorimeter tests at external heat fluxes of 35, 50 and 65 kW/m^2^. A linear fit was used to evaluate the HRP and corresponding y-cross point HRR_0_.

MC is frequently used as flame-retardant additive for PA6 formulations and acts through gas phase delusion by releasing large amounts of nitrogen [46,47,48]. Since no residue is formed during the decomposition process, the external heat impact is not limited over the course of a cone calorimeter test. Accordingly, high burning rates occurred, showing pHRR of 802 ± 141 kW/m^2^ for non-β-RC samples and 836 ± 67 kW/m^2^ for PA6 formulations containing filling degrees of 20 and 25 wt.% MC, respectively (Figure 11A).

The high pHRR characteristics compared to values measured for net PA6 can be attributed to an earlier reaction mechanism of MC. As already identified by thermal analysis (Section 3.2.2), MC decomposes at a temperature of ~335 °C, while the decomposition process of net PA6 starts at ~411 °C. When MC decomposes, large quantities of nitrogenous gases are generated. Due to the lower onset temperature, these gases are formed simultaneously with the decomposition processes of PA6, though in comparatively deeper layers. The nitrogen gases then migrate as bubbles through the material, creating a physical transport stream of nitrogen and polymeric fractions towards the surface [81]. Since large amounts of nitrogen gasses are released in the early heat exposure stages, the ignition time compared to net PA6 (~91 s) was delayed to 114 ± 3 s and 116 ± 6 s for PA6 formulations containing 20 and 25 wt.% MC, respectively. Once ignited, most of MC was consumed quickly, providing no sufficient delusion effect after ~4 min (Figure 11A). Some bubble formation accelerated the mass transfer of pyrolysis gases, providing large amounts of burnable gases and help explain the high pHRR values measured.

β-RC PA6 containing MC fractions tended to ignite earlier than non-β-RC samples (82 ± 9 s and 88 ± 5 s for 20 and 25 wt.% MC), but resulted in lower heat development over time. This was caused by a higher structural stability beyond the melting point of β-RC samples, which limited the movement of nitrogen gases generated in lower material layers. As a result, lower amounts of inert delusion gases were evaporated in early decomposition stages, reducing the associated flame inhibition effect and thus allowing earlier ignition. On the contrary, due to the limiting effect of radiation-β-RC samples, MC was consumed more slowly, so that lower amounts of nitrogen gases were released over a longer period of time. Thus, once a high level of heat development was reached, delusion caused flame inhibition effects reduced the overall burning rate, which resulted in a lower pHRR measured.

When PA6/MC formulations were tested at different external heat fluxes, the HRP revealed a substantially lower sensitivity of β-RC compared to non-β-RC samples (Figure 11B). Since the external heat flux dominates the intensity of temperature gradients present and thus controls the decomposition and mass loss rate over time, low heat fluxes of 35 kW/m^2^ induce a slower and more homogeneous heating process. This environment causes MC to decompose at much lower rates and can thus provide gas phase delusion over a longer period of time. Additionally, physical movement restrictions in β-RC samples are less dominant, since the decomposition zone is located close to the sample surface and bubble migration is rather limited to this area. Higher heat fluxes of 65 kW/m^2^ on the contrary induce high temperature gradients, whereas decomposition temperatures are quickly reached throughout the sample cross section. The b migration of pyrolysis and nitrogen gases is thus strong for non-β-RC samples and large MC fractions are quickly consumed, which in turn resulted in high pHRR measured. The β-RC samples revealed strong warpage, though still provided good physical stability. Bubble formation and pyrolysis gas transport from deeper layers is thus strongly limited, causing a lower mass loss rates and thus lower heat development overall.

#### 3.4.3. Cone Calorimeter Testing—EG

**Figure 12:**
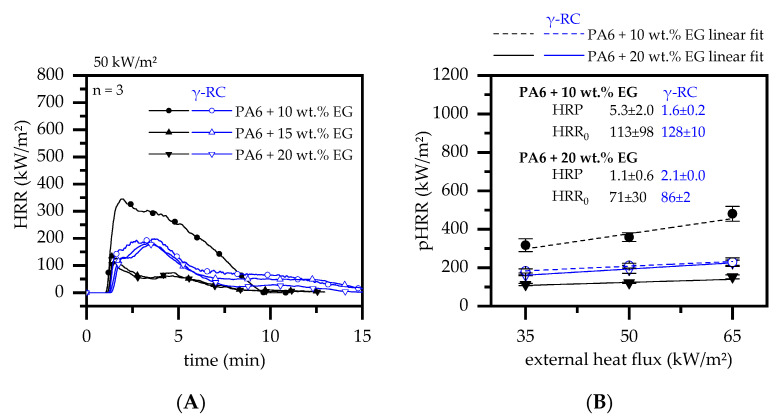
(**A**) HRR curves conducted by cone calorimeter testing for net and β-radiation β-RC PA6/EG formulations at 50 kW/m^2^. (**B**) pHRR conducted by cone calorimeter tests at external heat fluxes of 35, 50 and 65 kW/m^2^. A linear fit was used to evaluate the HRP and corresponding y-cross point HRR_0_.

When EG is integrated into a polymeric material, the flame retardant solely acts through the formation of a voluminous, thermally stable residue on the material’s surface. The residue predominantly provides a thermal isolation effect for lower polymer layers, which in turn limits the decomposition rate and thus the heat development over time. Cone calorimeter studies thus revealed a steady decrease in heat development, which is particularly efficient for higher filling degrees of 15 and 20 wt.% in non-β-RC samples (Figure 12A).

Accordingly, the pHRR dropped from 669 ± 12 kW/m^2^ for net PA6 to 24 ± 2 kW/m^2^ for PA6 containing 20 wt.% EG. β-RC PA6 showed a somewhat different behavior. Although β-RC PA6 formulations with low filling degrees of 10 wt.% EG showed superior properties in terms of heat generation over time, no further improvement was detected for higher filler contents. This effect can be attributed to two different mechanisms, whose dominance was shifted by the addition of different amounts of additive.

(1) Warpage and residue disruption: As described earlier for net PA6, β-RC samples tended to warp. Warpage caused the distance between the sample surface and cone heater to shrink, which increased the impact of heat. Additionally, cracks appeared within the growing graphite char residue due to the surface movement and were triggered through internal stress elimination. These cracks locally reduced the insulation performance and led to an increased impact of heat, subsequently causing accelerated pyrolysis gas production. This was particularly evident for formulations containing 15 and 20 wt.% EG.
(2) Mass transportation rate: Even though no shifts in the decomposition onset temperature could be detected, β-RC samples showed a higher physical stability throughout the burning process. This effect was attributed to a lack of low molecular melt pools, bubbling and random geometrical collapses, which constantly change the surface area during the burning process of non-β-RC samples. Particularly in the early burning stages, in which the decomposition zone of β-RC samples containing PA6 and EG occurred rather close to the surface, pyrolysis gas production was more limited. Since the flame retardant effect of EG strongly depends on the formation of residue and as the build up of residue is a somewhat time-consuming process, sufficient fire protection cannot be provided in early burning stages. However, since β-RC seemed to decelerate the mass loss rate development, EG particles were provided with a longer expansion time before ignition. The ignition was thus carried out slightly later.

The results show that the performance loss through warpage and cracking effects relative to decelerated mass loss rate development are particularly favorably balanced in formulations containing low EG filling degrees. This becomes clear when examining HRP (Figure 11B). β-RC PA6 formulations containing 10 wt.% EG exhibit a low HRP at an overall lower pHRR interception point HRR_0_ compared to non-β-RC samples. This implies a lower reaction to heat for this particular formulation and thus an overall superior fire inhibition. At higher filling degrees, EG provides a larger residual volume, which leads to an improved barrier effect already in earlier burning stages. The deceleration effect of the mass loss rate development provided by crosslinked structures thus loses its importance in the cumulated fire protection mechanism. Instead, warpage increases in dominance and prohibits further improvements.

#### 3.4.4. Cone Calorimeter Testing—AlPi

**Figure 13:**
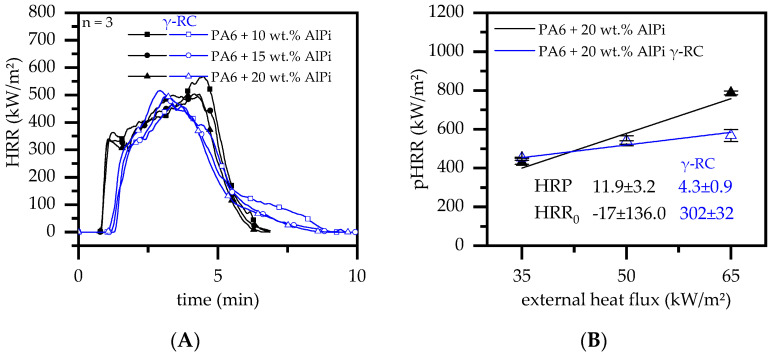
(**A**) HRR curves conducted by cone calorimeter testing for net and β-radiation β-RC PA6/AlPi formulations at 50 kW/m^2^. (**B**) pHRR conducted by cone calorimeter tests at external heat fluxes of 35, 50 and 65 kW/m^2^. A linear fit was used to evaluate the HRP and corresponding y-cross point HRR_0_.

Exolit 1230 is a phosphorous flame-retardant additive based on aluminum diethylphosphinat (AlPi). It acts predominantly by flame poisoning and some residue formation, which in turn limits the heat release rate over time in cone calorimeter testing setups. Since the residue formation of PA6/AlPi formulations is small, the flame-retarding performance in cone calorimeter environments is rather limited (Figure 13A). Compared to net PA6, non-β-RC samples ignited after around 53 s, which was followed by a steep increase in heat development. The pHRR measured was between 530 and 590 kW/m^2^. Only little improvements could be identified for higher filling degrees. Β-RC samples ignited somewhat later than non-β-RC samples at around 77–86 s. After ignition the heat development accelerated quickly, marking a slightly earlier but lower pHRR than measured for non-β-RC samples. Once the pHRR was reached, the subsequent development was characterized by a smaller decrease in the subsequent heat generation. Compared to non-β-RC samples, where a substantial amount of the polymer had already been burned at this stage, large non-burned material fractions were still physically available in β-RC samples. Thus, physical effects limiting the overall burning rate in β-RC samples appeared to be somewhat superior in cone calorimeter setups.

The characteristic differences evident for non- and β-RC samples were attributed to a serious of physical phenomena observed throughout the burning process. Shortly before ignition occurred for non-β-RC samples, the amount of pyrolysis gases sharply increased, which was heavily promoted by a large pyrolysis gas fraction rising as bubbles from deeper polymer layers. As a consequence, large quantities of flammable gases were already present at an early stage, thus explaining the early ignition time. The bubbling even accelerated after ignition occurred, irregularly perforating the surface during transition. Simultaneous residue formation close to the surface, provided by the phosphorus flame-retardant additive, seemed to be frequently interrupted and led to a delay in the buildup process of a seamless insulating layer. Accordingly, the corresponding protection effect was also delayed.

β-RC samples, on the contrary, did not show substantial bubble formation. Pyrolysis gas thus predominantly evaporated from areas close to the sample surface, releasing a lower amount of gaseous fuel which, as a consequence, delayed the ignition process. Additionally, residual formation progressed more homogeneously in the early burning stages, providing a better protection effect against external heat. With the ongoing burning process, warpage occurred for β-RC samples. Since the phosphorus residue has low physical flexibility, cracks appeared, causing the barrier effect to locally deteriorate. However, partial cracking did not seem to change the burning behavior. The overall curve was rather similar to that measured for β-RC net PA6. It was thus concluded that major changes given by crosslinking for PA6/AlPi formulations are predominantly due to the increased physical stability, which limited the fuel supply and thus the heat development over time. A similarity to the lower heat generation for net PA6 can be attributed to a combined effect of char formation and radical scavenger effects, which were not fundamentally different for non-β-RC and β-RC samples.

When evaluating the HRP calculated from cone calorimeter tests at 35, 50 and 65 kW/m^2^, β-RC samples revealed a lower sensitivity towards an external heat flux than non-β-RC samples (Figure 13B). Whereas the pHRR measured at lower external heat fluxes of 35 kW/m^2^ was essentially identical for non- and β-RC samples, an external heat impact of 65 kW/m^2^ resulted in a substantially higher pHRR for the non-β-RC sample. It was thus concluded that β-RC PA6/AlPi formulations, combining a limited release in pyrolysis gases, residue formation and flame poisoning effects act complementarily and are particularly superior in high heat impact scenarios.

#### 3.4.5. Cone Calorimeter Testing—IFR

**Figure 14:**
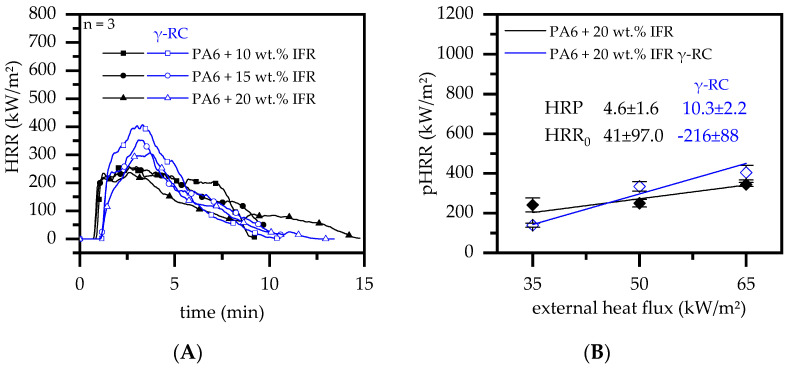
(**A**) HRR curves conducted by cone calorimeter testing for net and β-radiation β-RC PA6/IFR formulations at 50 kW/m^2^. (**B**) pHRR conducted by cone calorimeter tests at an external heat flux of 35, 50 and 65 kW/m^2^. A linear fit was used to evaluate the HRP and corresponding y-cross point HRR_0_.

Exolit OP 1314 (IFR) is a commercially available, synergistically modified flame-retardant additive mixture based on hypophosphite and melamine polyphosphate. The IFR has been reported to provide good flame-retarding properties by intumescence as well as radical savaging activities [77]. When IFR was incorporated into the PA6 matrix, cone calorimeter measurements revealed a large amount of char residue formed over the burning process, which drastically decreased the average heat release (Figure 14A). For non-β-RC samples, ignition occurred after 50–58 s. The subsequent heat development quickly proceeded, marking the pHRR between 250 and 287 kW/m^2^ shortly after ignition. A low pHRR and subsequent decline in the burning rate corresponds to a strong char formation, which developed rapidly in volume over time. β-RC samples ignited 75–77 s after the external heat source was applied. A subsequent steep increase in heat development was also evident, though the development speed towards higher heat release rates, eventually reaching a pHRR, seemed to be limited by growing char residue. Though, the pHRR was found to be substantially higher for β-RC samples, reaching values between 335 and 430 kW/m^2^. Higher filling degrees tended to decrease the heat development and resulted in notably lower pHRR.

As observed for a variety of other formulations tested within this study, β-RC samples suppressed the mass transport of pyrolysis gases originating from deeper layers, which caused strong bubbling in non-β-RC samples. The suppression of bubbling acted complementarily with the char residue formation, which in turn resulted in prolonged ignition times. With the ongoing burning process warpage becomes more dominant, physically bending the sample surface towards the cone heater. The physical bending process caused local residual cracking since the char residue did not provide the necessary flexibility to allow corresponding movement without damage. A closer proximity between the heater and surface additionally increased the heat impact, which in turn accelerated the burning process. Bubbling occurred for non-β-RC samples, shifting the ignition time towards lower temperatures. However, the strong bubble formation caused the char layer to quickly expand, resulting in a voluminous char residue already in very early burning stages. As a consequence, a lower pHRR and an overall low heat development were measured for non-β-RC samples.

Radiation β-RC samples showed more sensitive heat responses to the different external heat fluxes applied (Figure 14B). Low external heat fluxes tended to reveal lower pHRR than non-β-RC samples, whereas heat fluxes of 65 kW/m^2^ showed the opposite characteristic. This is very interesting, since the effect turned out to be the reverse of the HRP measured in PA6/AlPi samples.

#### 3.4.6. Summary—Cone Calorimeter Testing

A wide variety of flame-retardant additives providing different flame inhibition mechanisms have been studied within in non-β-RC and β-RC samples based on PA6 and tested in a cone calorimeter setup. Radiation crosslinking appeared to invoke a series of dominant, non-chemical changes within the burning behavior measured in cone calorimeter tests that physically altered the flame inhibition effect over time. The resulting effect was found to be entirely dependent on the individual flame inhibition mechanism present. The main alterations identified can be summarized as follows. Relevant key figures are listed in Table 3:

(1) β-RC samples tended to sufficiently suppress the mass transport of pyrolysis gases that originated from lower polymer layers, which was otherwise evident through the strong bubbling in non-crosslinked samples. The limited mass transport of pyrolysis gases was not only relevant in the early burning stages, but rather reduced the overall heat development over the burning process. This effect can be attributed to the higher structural stability provided by the three-dimensional crosslinked network.
(2) Chemical reactions triggered by flame-retardant additives that react with the matrix polymer and form a char residue predominantly occur between the melting point and the decomposition onset of the polymer. Since the three-dimensional crosslinked network physically stabilizes the polymeric melt until decomposition occurs, the freedom in movement as well as the amount of reactive and unbound polymer fractions is more limited than in non-crosslinked samples.
(3) The ongoing burning process caused warpage for all tested formulations. The physical deformation caused the proximity between the sample surface and cone heater to shrink, which resulted in a higher heat impact and accelerated the burning process. Additionally, when flame inhibition effects provided residual formation of any kind, the residual characteristics tended to deteriorate as a consequence of the physical deformation.

Since the ignition time did not change for non- and β-RC net PA6 samples, a shift towards lower or higher ignition times must be related to interactions between the additive and the polymeric matrix. MC, as a flame-retardant mechanism, does not interact with the polymeric matrix, though it depends on the release rate of nitrogen gases in order to shift the ignition onset time. Thus, since the release rate was high for non-crosslinked samples and low for crosslinked samples, the ignition time changed accordingly. EG also does not react with the matrix polymer. The flame-retarding effect is strictly related to the built-up speed and residual volume, providing a sufficient thermal barrier. In early burning stages, a sufficient residue has not yet been built, with no flame inhibition effect. Since the mass loss rate was more limited in crosslinked materials and the pyrolysis gas vaporization rate is lower due to the network effect, the expansion process has more time to built-up until a critical mass loss rate is reached. At the time a sufficient mass loss rate for ignition is reached, a larger residual fraction has been built, providing an improved thermal isolation effect. The phosphorus-based flame-retardant additives Exolit 1230 (AlPi) and Exolit 1314 (IFR) also showed a delay in ignition time. The corresponding effect was attributed to similar effects reported for crosslinked samples, limiting the polymeric fuel supply in the early burning stages.

**Figure 15:**
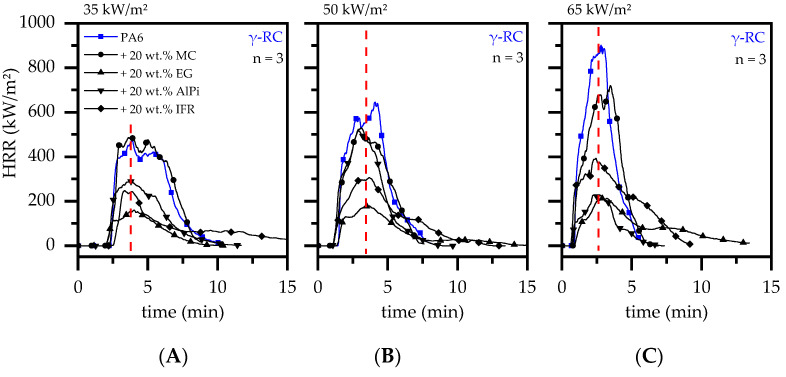
Comparative illustrations of cone calorimeter results conducted at (**A**) 35 kW/m^2^, (**B**) 50 kW/m^2^ and (**C**) 65 kW/m^2^ for a selection of β-RC samples.

However, the subsequent heat development showed somewhat different characteristics. Even though the evaporation rate in crosslinked samples seemed to also be restricted in later burning stages, heat release curves exhibited a similar increases and decreases. This effect was identified to be predominantly caused by warpage, creating a systematic fingerprint pattern evident in cone calorimeter curves (Figure 15A–C). Apparently, the time to pHRR was more or less identical for all crosslinked materials tested, which suggests that the measured pHRR are strongly influenced by the physical deformation. It is thus concluded that the natural pHRR without warpage would be lower than the values measured in cone calorimeter studies.

### 3.5. Fire Testing—LOI and Vertical UL-94

**Figure 16:**
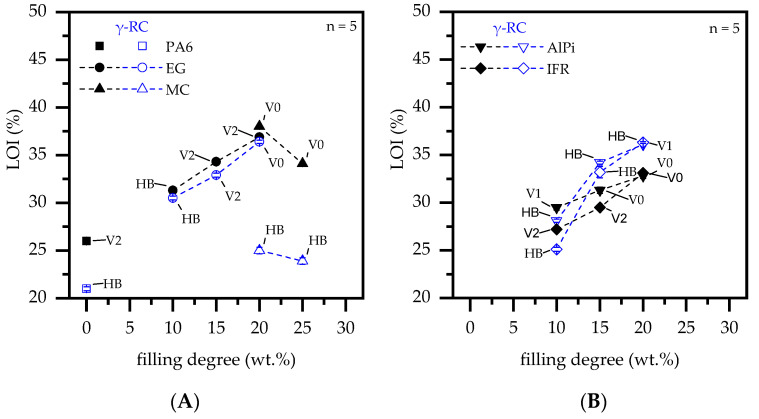
LOI and UL-94 V testing results for 1 mm-thick samples. (**A**) PA6, PA6/EG and PA6/MC formulations. (**B**) PA6/AlPi and PA6/IFR.

UL-94 and LOI burning tests are sensitive to the low melt viscosity of the tested polymer, which provides a physical transportation mode of molten material from the burning zone towards non-burning areas. Since the testing flame is comparatively small and the appearance of a melt flow removes a substantial amount of polymeric material covered by the flame, LOI values and UL-94 classifications tend to improve. This behavior allowed non-β-RC PA6 to achieve a V2 classification due to burn-melt dripping and a comparatively high LOI value of 26% (Figure 16A). The ability to remove polymeric material fractions from the burning zone by melt flow is prevented in β-RC materials. No melt dripping occurred in β-RC PA6, leading to an UL-94 HB classification and an LOI value of 21%.

When incorporated into a PA6 matrix, MC further reduces the melt flow while additionally evaporating nitrogen gases. This combines melt flow and dilution effects, improving the flame inhibition behavior observed in LOI and UL-94 burning tests. Accordingly, a V0 classification was achieved for filling degrees of 20 and 25 wt.% MC, whereas the LOI value slightly decreased from 38% to 34% when higher MC filling degrees were present. Similar values have been reported in the literature [48]. β-RC PA6 containing MC substantially reduced the melt flow behavior and limited the evaporations rate of nitrogen gases. As a consequence, UL-94 tests did not result in any classification, whereas some improvements were indicated in LOI test results. The values are 25% and 24% for 20 and 25 wt.% MC, respectively, which were slightly higher compared to the β-RC PA6 reference.

Non-β-RC formulations based on PA6/EG showed a steady increase in the oxygen index for higher filling degrees and revealed V2 classifications for filler contents of 15 and 20 wt.%. A V2 classification was reached due to burn breaking and subsequent flame extinguishing at the remaining sample. Similar values have been reported in the literature [50,51,52]. Materials which exceed oxygen indices of >35% usually achieve a V0 classification in UL-94 V fire tests. In the case of expandable graphite, a high thermal conductivity provides an improved heat distortion effect, which has a positive effect on LOI testing results. A corresponding study addressing this issue can be found in [79]. β-RC formulations containing EG showed a comparable LOI development. Larger EG fractions present within the formulation improved the residual formation, which resulted in a steady increase in LOI values. A V0 classification could be achieved for β-RC formulations containing 20 wt.% EG. When burn breaking occurred in non-β-RC PA6/EG formulations, a substantial fraction of the already built-up residue erupted, leaving a non-protected hole in the remaining residue. β-RC provided an improved melt stability in PA6/EG formulations, which stabilized initially built-up residue and efficiently suppressed burn breaking. Correspondingly, a higher melt stability was found to improve the achieved flame retardancy effect of β-RC PA6/EG formulations in vertical UL-94 burning tests, resulting in superior classifications being achieved.

Formulations containing AlPi or IFR also revealed increasing LOI values for higher additive contents present (Figure 16B). For non-β-RC samples, formulations containing AlPi and IFR LOI values increased to 30, 31, and 33% and 27, 30, and 36% for filling degrees of 10, 15 and 20 wt.%, respectively. A V0 classification could be achieved for filling degrees of 15 and 20 wt.% in PA6/AlPi and 20 wt.% in PA6/IFR formulations. β-RC formulations were also found to increase the LOI value measured, achieving 2–3% higher values when compared to non-β-RC formulations. The lower flammability tendency of β-RC formulations fits well to the effects also found in cone calorimeter measurements. The lower evaporation rates of pyrolysis gases limited the fuel supply and thus prolonged ignition times in cone calorimeter tests. In LOI testing setups, a similar effect was evident, as evidenced by the higher LOI values. A lower fuel supply combined with the flame inhibition mechanisms given by the phosphorous flame-retardant additives only allowed ignition at higher oxygen levels present. However, UL-94 classifications turned to deteriorate for β-RC formulations, only resulting in a V1 classification for PA6 containing 20 wt.% of IFR. β-RC formulations tended to deform during and after flame exposure, with the lower end of the specimen gradually curling upwards. Since no lasting ignition occurred after the first exposure time for samples containing >15 wt.% of either phosphorous additive, the deformation caused a larger sample fraction to be exposed during the second ignition attempt. A final ignition occurred after the second exposure time. During the burning process gradual curling proceeded and no burn dripping/breaking occurred until the sample is fully burned. Residual formation and flame inhibition effect cannot compete with the quick flame spread caused by physical curing. All discussed key figures have been summarized in Table 4.

### 3.6. Residue Analysis—Cone Calorimeter

**Figure 17:**
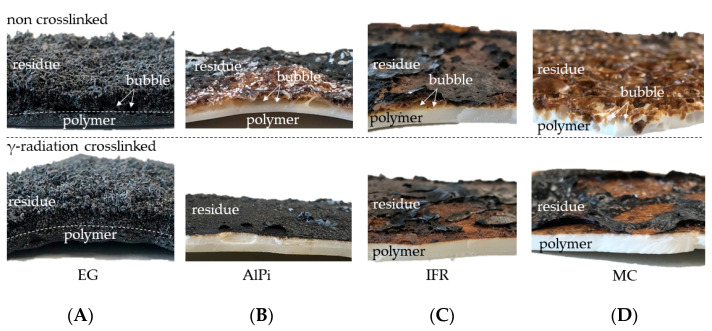
β-RC and non-β-RC samples extracted from cone calorimeter tests (50 kW/m^2^) after 120 s. (**A**) PA6 + 20 wt.% EG; (**B**) PA6 + 20 wt.% AlPi; (**C**) PA6 + 20 wt.% IFR; (**D**) PA6 + 25 wt.% MC.

In order to gain a better understanding of the different burning behaviors evident for non-β-RC and β-RC formulations, cone calorimeter samples were analyzed 120 s after ignition as well as after complete combustion. Samples were thus removed after 120 s, extinguished and cooled. After complete solidification, the samples were broken. Images representing the fragments cross-sections are presented in Figure 17A–D.

As discussed earlier in the cone calorimeter results section (Section 3.4), all β-RC samples were characterized by increased warpage and significantly reduced bubble formation during cone calorimeter testing. Both effects were visually evident within samples removed after 120 s. Since the presented cross-sections shown in Figure 17 are broken fragments, warpage is visually not as evident. The images are focused on the visualization of bubble formation, which underlines the fuel transportation limits as well as the in-depth combustion progress.

Non-β-RC samples show substantial bubble formation, which is particularly evident in areas close to the surface. The decomposition process is also visible over a larger cross-section, suggesting a larger mass transfer of pyrolysis gases towards the surface. On the contrary, residual formation visible in β-RC materials is rather restricted to near-surface areas. No color changes occurred in deeper layers, signaling ongoing decomposition processes. Moreover, residual formation seemed to be more homogeneous, since the absence of bubble formation caused no irregularities.

**Figure 18:**
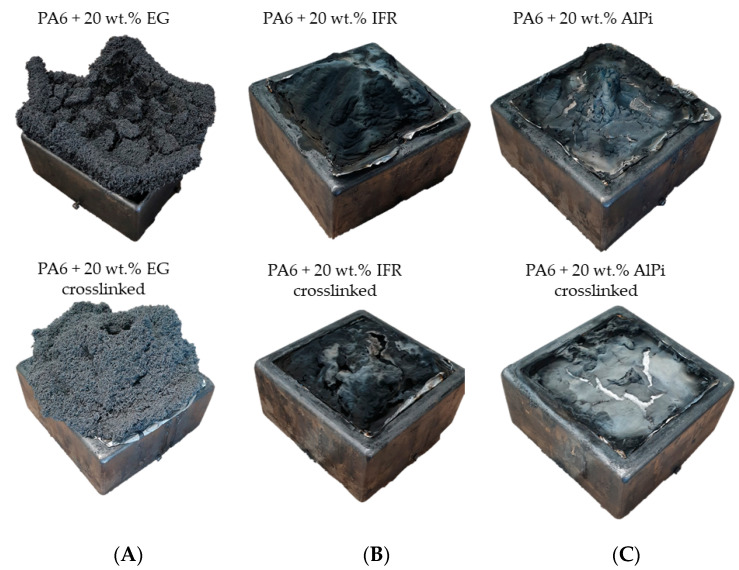
Char residues of non-β-RC and β-RC materials after full decomposition within cone calorimeter tests (50 kW/m^2^). (**A**) PA6/EG, (**B**) PA6/IFR, and (**C**) PA6/AlPi.

Figure 18 shows residual images taken of cone calorimeter samples after complete combustion. Net PA6 and PA6 containing MC as flame-retardant additive did not show any residual fractions and are thus not presented. The largest residual fractions were observed for formulations containing EG (Figure 18A). Under the cone calorimeter test conditions, EG does not decompose, so that its total weight fraction remains as voluminous residue.

β-RC and non-β-RC residues appeared geometrically different, revealing a predominantly flat or conical geometry. The conical geometry can be attributed to proceeding warpage, whereas the sample physically deformed, warping central sample areas closer to the cone heater. EG particles underwent deformation, laterally tilted and formed an opening in the core area. The central opening provided a favorable exit point for rising pyrolysis gases, which consequently caused the flaming combustion to be predominantly located above the opening.

Phosphorus flame-retardant additives act partly by flame poisoning and residual formation. The char residue is formed by a complex cascade of chemical reactions, which thus physically proceeds over time as part of the polymeric and additive decomposition process. The most voluminous residue was formed from interactions between the intumescence flame-retardant additive and the polymeric matrix, which was found to be visually and chemically identical for non-β-RC and β-RC formulations (Figure 18B). However, cracks and holes were more dominant in residues formed in β-RC samples, which was attributed to the physical deformation process caused by warpage. The char residue provided little flexibility when exposed to physical stress, which, as a consequence, caused local cracking to occur. PA6/AlPi formulations showed less residue formation (Figure 18C). Cone calorimeter measurements, as discussed in Section 3.4, indicated substantially less char formation compared to PA6/IFR formulations, which is also reflected in the lower amount of residue. Since the char formation was substantially smaller and no chemical differences were identified between non-β-RC and β-RC formulations, changes along the built-up process did not seem to have a dominant impact on the fire behavior in cone calorimeter tests.

## 4. Discussion

The fire behaviors of non-β-RC and β-RC PA6 modified by four commercially available flame-retardant additives have been investigated. This study focused on the identification of alterations in the fire behavior that could be attributed to radiation crosslinking and ultimately led to changes in fire protection classifications in a variety of different fire testing scenarios. Thermal analysis, FITR gas analysis, and dynamic mechanical analysis have been conducted in order to gain fundamental knowledge about material properties. Cone calorimeter tests, limiting oxygen index and UL-94 tests as well as residual analysis have been performed to identify changes within the burning characteristics.

For the given flame-retardant formulations tested, the study indicated no chemical changes within the decomposition pathways. Differences that have been identified to alter the burning behavior in non-β-RC and β-RC materials within this study were thus solely attributed to a series of physical phenomena. However, since other studies have reported the chemical changes of decomposition characteristics within other polymeric material systems that effectively altered the burning behavior [16,17,18], a strictly physical interpretation is not always permissible and must be determined if it is appropriate on an individual basis. The physical phenomena that were observed alter the burning behavior due to radiation crosslinking are listed as follows:

- The limited physical mass transport of pyrolysis gasses through the material cross section (e.g., bubbling).
- The limited decomposition depth to near surface areas.
- Anti-dripping
- Physical stability at elevated temperatures; no structural collapse occurred.
- Strong warpage.

Regardless of the formulations tested, all phenomena were clearly shown to change burning characteristics. However, the impact on the fire behavior strongly varied in terms of dominance, which is not only dependent on the flame-retardant additive, but also the filling degree present.

The limited physical mass transport given by the three-dimensional network of RC samples was found to be counterproductive for the flame-retarding mechanism of MC. MC mainly acts through the release of large nitrogen gas fractions in order to dilute burnable pyrolysis gasses. As a result, no ignition or prolonged ignition times occur. RC networks suppressed this mechanism, which resulted in declined fire inhibition effects in the cone calorimeter, UL-94 and LOI fire testing. Formulations containing EG, on the other hand, benefitted from lower mass loss rates, particularly in the earlier burning stages. Longer ignition times provided a prolonged residual built-up time, which resulted in an improved barrier effect once ignition occurred. This was found to be particularly beneficial in UL-94 fire testing environments, whereas in cone calorimeter tests only low filling degrees of 10 wt.% provided a substantial improvement compared to non-β-RC samples. The phosphorus-containing flame-retardant additives AlPi and IFR both resulted in lower performances, as measured using UL-94 tests, but appeared to provide superior characteristics in LOI testing environments. In the cone calorimeter tests, the ignition times were prolonged for β-RC samples, though major improvements could not be identified.

Warpage has been reported to be a major phenomenon-that influences the burning behavior of β-RC materials [24]. Fire scenarios are characterized by a non-uniform heat exposure, causing strong temperature gradients within the exposed material. Since the three-dimensional network in RC materials provides structural stability at elevated temperatures, thermal elongation causes asymmetric geometrical deformation, usually directed towards the external heat source. As a result, the heat impact was increased and the burning process accelerated. Cone calorimeter tests revealed a general curve characteristic, exhibiting the time for pHRR to occur, which was identical for all tested formulations and heater capacities adjusted.

## 5. Conclusions

RC provides a commercial possibility to adjust specific material properties, which has been mainly utilized to improve the performance of chemical, mechanical, thermo-mechanical and tribological properties [3,4,5,6,7]. Fire safety issues, however, have not yet been extensively addressed, even though RC provides a variety of interesting characteristics that might be generally beneficial for fire safety efforts. This includes the restrictive mass transport of pyrolysis gases towards a flame, a lower mesophase depth, shorter ignition times and anti-dripping properties. Since most flame-retardant additives are used in non-RC materials, commercially available additives are designed to provide highly effective solutions to achieve specific fire safety classification, but do not necessarily work sufficiently in RC materials. This is particularly evident for the formulations tested within this study. Both the phosphorus flame-retardant additives AlPi and IFR as well as MC provided excellent flame inhibition properties in UL-94 and LOI fire tests for non-β-RC PA6 formulations. However, comparable formulations that were β-RC did not provide sufficient UL-94 classification. Only PA6 containing EG indicated an improvement in UL-94 classification to V0 in β-RC compared to V2 measured for non-β-RC samples.

Further research is needed in order to develop flame-retardant systems specifically designed for RC materials. New material strategies capable of exploiting RC advantages and counteracting/balancing disadvantages are required. These might include rapid charring close to the surface or flexible residue formation, counteracting warpage-induced residual breakage.

## Figures and Tables

**Table 1:** Materials and recipes.

Materials	Abr.	Filling Degree (wt.%)
Polyamide 6 (B27E)	PA6	matrix
melamine cyanurat (MC50)	MC	20, 25
expandable graphite (GHL PX 95 HT)	EG	10, 15, 20
Aluminum (diethyl-)polyphosphinat (Exolit OP 1230)	AlPi	10, 15, 20
intumescent combination (Exolit OP 1314)	IFR	10, 15, 20
Triallyl Isocyanurate TAIC (TAICROS PA6 60); 40% TAIC/60% PA6	TAIC	3

**Table 2:** TGA measurement—summary.

PA6 wt.%	MC wt.%	EG wt.%	AlPi wt.%	IFR wt.%	TAIC wt.% (RC)	T_99%_ Onset °C	T_97%_ Onset °C	T_90%_ Onset °C	DTG-Peak °C/min	Residue %	Vyazovkin [27] kJ/mol	Ozawa [28] kJ/mol
100						351 ± 2	399 ± 1	425 ± 1	479 ± 2	1.7 ± 0.0	195	218
97					3	366 ± 2	400 ± 1	428 ± 2	468 ± 2	0.9 ± 0.0	187	210
80	20					320 ± 1	335 ± 2	355 ± 2	357 ± 2; 466 ± 2	1.0 ± 0.0	175, 148	194, 170
77	20				3	320 ± 2	335 ± 2	355 ± 2	357 ± 2; 466 ± 2	1.1 ± 0.4	201, 155	221, 177
80		20				333 ± 2	359 ± 2	404 ± 1	460 ± 2	23.4 ± 1.0	183	206
77		20			3	350 ± 1	376 ± 1	412 ± 2	460 ± 2	26.3 ± 2.0	193	216
80			20			357 ± 2	390 ± 2	419 ± 2	447 ± 1	3.8 ± 0.3	200	222
77			20		3	376 ± 2	397 ± 2	419 ± 2	454 ± 2	2.5 ± 0.1	212	234
80				20		369 ± 1	385 ± 2	409 ± 1	445 ± 1	5.5 ± 0.5	207	230
77				20	3	369 ± 2	385 ± 2	409 ± 2	446 ± 2	5.7 ± 0.3	204	227

**Table 3:** Cone calorimeter results measured at an external heat flux of 50 kW/m^2^—summary.

PA6 wt.%	MC50 wt.%	EG wt.%	Exo 1230 wt.%	Exo 1314 wt.%	TAIC wt.%	t_ign_ s	pHRR kW/m^2^	THE MJ/m^2^	TSP m^2^
100						91 ± 4	778 ± 45	134 ± 3	7.4 ± 0.2
97					3	91 ± 1	669 ± 12	128 ± 2	8.0 ± 0.1
80	20					114 ± 3	802 ± 141	112 ± 5	6.6 ± 0.5
75	25					116 ± 6	836 ± 67	119 ± 4	7.2 ± 0.5
77	20				3	82 ± 9	572 ± 27	112 ± 7	7.1 ± 0.3
72	25				3	88 ± 5	549 ± 40	119 ± 3	6.8 ± 0.8
90		10				71 ± 1	359 ± 23	103 ± 2	7.5 ± 0.1
85		15				72 ± 3	167 ± 29	26 ± 2	2.2 ± 1.3
80		20				77 ± 1	122 ± 3	24 ± 2	1.6 ± 0.2
87		10			3	79 ± 2	211 ± 13	74 ± 5	3.3 ± 0.5
82		15			3	85 ± 3	195 ± 20	62 ± 3	2.5 ± 0.1
77		20			3	90 ± 2	191 ± 21	48 ± 0	1.9 ± 0.3
90			10			54 ± 4	587 ± 29	119 ± 1	23.5 ± 0.1
85			15			54 ± 1	531 ± 45	111 ± 5	26.5 ± 1.2
80			20			53 ± 2	532 ± 9	109 ± 2	26.6 ± 1.1
87			10		3	86 ± 4	482 ± 8	104 ± 1	23.6 ± 0.1
82			15		3	85 ± 8	518 ± 26	96 ± 1	24.8 ± 0.9
77			20		3	77 ± 5	540 ± 25	99 ± 3	26.3 ± 0.6
90				10		58 ± 2	287 ± 8	94 ± 7	22.8 ± 0.7
85				15		50 ± 1	276 ± 10	98 ± 5	24.0 ± 0.6
80				20		52 ± 2	250 ± 20	92 ± 5	21.6 ± 1.3
87				10	3	77 ± 2	430 ± 54	99 ± 8	22.6 ± 0.5
82				15	3	75 ± 3	370 ± 20	94 ± 2	22.9 ± 0.4
77				20	3	77 ± 5	335 ± 24	84 ± 11	22.8 ± 0.7

**Table 4:** LOI and vertical UL-94 burning test results—summary.

Material	β-RC	LOI	UL-94	t1 + t2	Burn Dripping
PA6	no	26	V2	3 s	yes
	yes	21	HB	-	no
PA6 + 20, 25 wt.% MC	no	38, 34	V0, V0	3 s, 6 s	no, no
	yes	25, 24	HB, HB	-, -	no, no
PA6 + 10, 15, 20 wt.% EG	no	31, 34, 37	HB, V2, V2	-, 8 s, 8 s	yes, yes, yes
	yes	30, 33, 36	HB, V2, V0	-, 2 s, 2 s	yes, yes, no
PA6 + 10, 15, 20 wt.% AlPi	no	30, 31, 33	V1, V0, V0	14 s, 4s, 3 s	no, no, no
	yes	28, 34, 36	HB, HB, HB	-, -, -	no, no, no
PA6 + 10, 15, 20 wt.% IFR	no	27, 30, 33	V2, V2, V0	11s, 8s, 6 s	yes, yes, no
	yes	25, 33, 36	HB, HB, V1	-, -, 14 s	yes, yes, no
